# *Salmonella* Typhimurium disrupts Sirt1/AMPK checkpoint control of mTOR to impair autophagy

**DOI:** 10.1371/journal.ppat.1006227

**Published:** 2017-02-13

**Authors:** Raja Ganesan, Nina Judith Hos, Saray Gutierrez, Julia Fischer, Joanna Magdalena Stepek, Evmorphia Daglidu, Martin Krönke, Nirmal Robinson

**Affiliations:** 1 Institute for Medical Microbiology, Immunology and Hygiene, University of Cologne, Cologne, Germany; 2 Cologne Cluster of Excellence in Cellular Stress Responses in Aging-Associated Diseases (CECAD), University of Cologne, Cologne, Germany; 3 German Center for Infection Research (DZIF), Cologne, Germany; 4 First Department of Internal Medicine, University of Cologne, Cologne, Germany; University of Michigan Medical School, UNITED STATES

## Abstract

During intracellular infections, autophagy significantly contributes to the elimination of pathogens, regulation of pro-inflammatory signaling, secretion of immune mediators and in coordinating the adaptive immune system. Intracellular pathogens such as *S*. Typhimurium have evolved mechanisms to circumvent autophagy. However, the regulatory mechanisms targeted by *S*. Typhimurium to modulate autophagy have not been fully resolved. Here we report that cytosolic energy loss during *S*. Typhimurium infection triggers transient activation of AMPK, an important checkpoint of mTOR activity and autophagy. The activation of AMPK is regulated by LKB1 in a cytosolic complex containing Sirt1 and LKB1, where Sirt1 is required for deacetylation and subsequent activation of LKB1. *S*. Typhimurium infection targets Sirt1, LKB1 and AMPK to lysosomes for rapid degradation resulting in the disruption of the AMPK-mediated regulation of mTOR and autophagy. The degradation of cytosolic Sirt1/LKB1/AMPK complex was not observed with two mutant strains of *S*. Typhimurium, *ΔssrB* and *ΔssaV*, both compromising the pathogenicity island 2 (SPI2). The results highlight virulence factor-dependent degradation of host cell proteins as a previously unrecognized strategy of *S*. Typhimurium to evade autophagy.

## Author summary

*S*. Typhimurium is a facultative intracellular pathogen which uses its type III secretion system to avoid cell-autonomous defense mechanisms such as autophagy. Here we show that *S*. Typhimurium induces energy depletion resulting in an early but transient activation of AMPK and autophagy. *Salmonella* virulence factors target Sirt1/LKB1/AMPK for lysosomal degradation, which enables sustained mTOR-activation and inhibition of autophagy. Activation of mTOR establishes a molecular feedback loop that enhances lysosomal degradation of Sirt1/LKB1/AMPK.

## Introduction

*Salmonella enterica* serovar Typhimurium (*S*. Typhimurium) is a facultative intracellular Gram-negative pathogen, which causes gastroenteritis in humans and typhoid like disease in mice. The virulence factors of *S*. Typhimurium are organized in two gene clusters called Salmonella Pathogenicity Island 1 and 2 (SPI1 and SPI2), which encode two distinct, type-3 secretion systems (T3SS). The effector proteins of SPI1 are critically important for invading non-phagocytic cells. SPI2 dependent effector proteins enable the pathogen to create a niche in the salmonella containing vacuole (SCV) for replication, which is important for intracellular survival of the pathogen [1]. Internalized pathogens are subjected to xenophagy, a special form of autophagy that targets intracellular pathogens for lysosomal degradation. Autophagy is an evolutionarily conserved process, which is essential in maintaining cellular homeostasis by eliminating damaged organelles for recycling. Hence, autophagy is vital in promoting cell survival under various stressful conditions, such as pathogen infection, nutrient and growth factor deprivation, or mitochondrial and endoplasmic reticulum stress. Autophagy occurs at basal levels in cells, but is upregulated upon stress such as pathogen invasion [2]. It also contributes to the elimination of many intracellular pathogens including *Mycobacterium tuberculosis* [3]. In contrast, *S*. Typhimurium-induced autophagy enables bacteria to obtain nutrients and replicate [4]. Various receptors such as optineurin [5], galectin8 [6], NDP52 [7] and ubiquitin modifiers such as FAT10 [8] have been shown to assist in targeting cytosolic *S*. Typhimurium into the autophagosome.

Autophagy is controlled by mammalian target of rapamycin (mTOR) signaling pathway. mTOR senses nutrient availability and metabolic changes in the cell. Activation of mTOR results in the formation of multiprotein complexes mTORC1 and mTORC2 [9]. Inhibition of mTORC1 increases autophagy, whereas its activation results in the cessation of autophagy [10]. It has been reported that *S*. Typhimurium rapidly depletes intracellular amino acid pools, which results in transient inhibition of mTORC1 and activation of autophagy. It is important to note that *S*. Typhimurium counteracts autophagy by activating mTORC1 [11]. However, the interplay of molecular signals that control mTOR activity and promote autophagy in *S*. Typhimurium infected cells remains elusive.

*S*. Typhimurium evades phagosome degradation associated with different forms of cell death including apoptosis, pyroptosis and necroptosis [12,13]. In macrophages, *S*. Typhimurium induces a type-I-Interferon-mediated, energy-depleting necroptotic cell death, which results in the loss of host’s resistance and tolerance against the pathogen [14]. Adenosine monophosphate kinase (AMPK) is a crucial intracellular energy sensor that is activated upon decline in ATP and increase of the AMP/ATP ratio. Activation of AMPK restores energy levels by enhancing mitochondrial biogenesis and autophagy [15]. AMPK activation is initiated upon binding of AMP to AMPK, which allows the upstream kinase, liver kinase B1 (LKB1) to phosphorylate AMPK [16]. The ability of LKB1 to phosphorylate AMPK is dependent on the deacetylation of its lysine residue by Sirtuin-1 (Sirt1) [17]. Sirt1 belongs to the family of lysine deacetylases and plays an important role in the activation of AMPK [18]. Sirt1 is predominantly localized in the nucleus yet translocates to the cytoplasm in response to the PI3K-AKT signaling pathway [19]. Sirt1 mainly exerts its cell autonomous functions by regulating various transcription factors such as p53, FOXO1, FOXO3A and NF-κB [20] in the nucleus. Sirt1 regulates cellular repair mechanisms such as mitochondrial biogenesis and autophagy [21]. It governs the formation of autophagic vacuoles by deacetylating the Atg5, Atg7 and Atg8 (LC3) complex[22]. In addition, Sirt1-dependent activation of AMPK leads to inhibition of mTOR, which also propels autophagy [18,23]. Notably, AMPK provides NAD+ for the activity of Sirt1 thereby establishing a positive feedback loop [24], which is expected to result in prolonged autophagy. However, little is understood about the role of Sirt1 in pathogen-induced autophagy.

In this study, we delineate how *S*. Typhimurium disrupts the Sirt1/LKB1/AMPK circuit acting as an mTOR checkpoint control. Specifically, we show that *S*. Typhimurium infection induces lysosomal degradation of Sirt1, LKB1, and AMPK, which unleashes mTOR and eventually results in impaired autophagy. The results of this study identify the Sirt1/LKB1/AMPK complex as a previously unrecognized target for SPI2 encoded effector proteins by which *Salmonella* manipulates the important checkpoint mTOR to compromise autophagic host cell defense mechanisms.

## Results

### *S*. Typhimurium infection results in energy depletion and transient activation of AMPK

**Fig 1:**
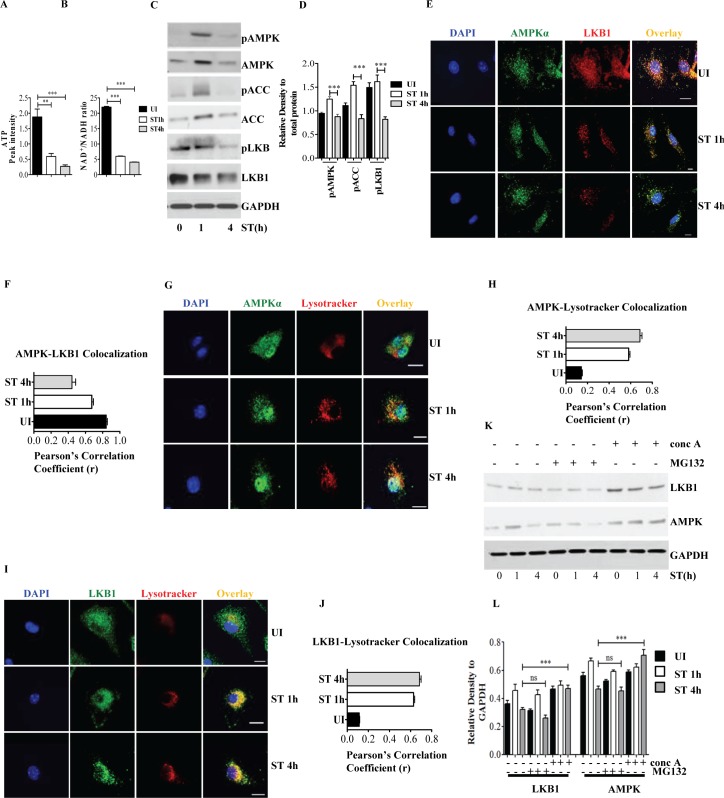
*S*. Typhimurium infection results in energy loss and transient activation of AMPK. **(A)** ATP levels in BMDMs upon *S*. Typhimurium was analyzed by mass spectrometry and the mass peak intensity is depicted in the graph as mean ± SEM, ***p≤0.001 (n = 6). **(B)** Intracellular NAD^+^ and NADH levels form uninfected and *S*. Typhimurium-infected BMDMs were measured using NAD+/NADH assay kit. Bar graphs are expressed as mean ± SEM, ***p≤0.001 (n = 3). **(C)** Immunoblot analysis of AMPK, ACC and LKB1 expression upon *S*. Typhimurium infection in BMDMs cells. **(D)** Mean densitometric analysis of immunoblots is shown. Data are representative of 3 independent experiments. Bar graphs are expressed as mean ± SEM, ***p≤0.001 and **p≤0.01. **(E)** Confocal image showing AMPK-LKB1 (n = 4). (**F**) Pearson’s correlation coefficient of AMPK with LKB1 analyzed from 50 regions of interest (ROI). **(G)** AMPK-LysoTracker Red co-localization (n = 4). **(H)** Pearson’s correlation coefficient of AMPK with LysoTracker Red analyzed from 50 ROIs. **(I)** LKB1-LysoTracker Red co-localization in BMDMs upon *S*. Typhimurium infection n = 3. (**J**) Pearson’s correlation coefficient of LKB1-LAMP1 co-localization calculated by measuring minimum of 50 ROI using olympus fluoview fv1000 software. Scale bar = 10μm for microscopy images. **(K)** Total AMPK and LKB1 expression upon *S*. Typhimurium infection in BMDMs treated with concanamycinA (concA) or MG132. Western blots are representative of three experiments. **(L)** Mean densitometric data of protein expression were analyzed using NIH Image J software. Bar graphs are expressed as mean ± SEM, ns non-significant, ***p≤0.001 and **p≤0.01.

Previously we had shown that *S*. Typhimurium induces necrotic cell death in macrophages [14]. Because this form of cell death is correlated with energy depletion we began to investigate specific markers of metabolic energy in *S*. Typhimurium-infected bone marrow-derived macrophages (BMDMs). Indeed, ATP as well as NAD+ levels dropped in macrophages over time upon *S*. Typhimurium infection **(Fig 1A and 1B and S1A Fig)**. Intracellular decline in levels of ATP and NAD+ trigger the activation of adenosine monophosphate kinase (AMPK) [25]. Despite sustained low levels of ATP and NAD+ in *S*. Typhimurium-infected macrophages, AMPK was only transiently activated at 1h and then declined to basal level at 4h as inferred from the phosphorylation of AMPK and acetyl coA carboxylase (ACC), a *bona fide* substrate of AMPK **(Fig 1C and 1D)**. LKB1 activates AMPK [26], therefore we asked if the biphasic AMPK activation is under the control of LKB1. Interestingly, phosphorylated and non-phosphorylated forms of LKB1 were downregulated upon infection **(Fig 1C and 1D)**. Consistently, microscopical examinations revealed that both abundance and co-localization of LKB1 with AMPK was reduced at 4h post infection **(Fig 1E)**. Pearson’s correlation coefficient analysis confirmed decreased co-localization **(Fig 1F)**. *S*. Typhimurium infection also induced increased co-localization of AMPK **(Fig 1G and 1H)** and LKB1 with LysoTracker Red **(Fig 1I and 1J)** and LAMP1 (Lysosome associated membrane protein-1) (**S1B–S1E Fig**) suggesting that AMPK and LKB1 were degraded in lysosomes. We confirmed the lysosomal degradation of AMPK and LKB1 **(Fig 1K and 1L)** by inhibiting lysosomal activity using concanamycin A, which also prevented the degradation of p62 a target of lysosomal degradation **(S1F Fig)**. Degradation of AMPK and LKB1 was dependent on the virulence of *S*. Typhimurium because the heat-killed *S*. Typhimurium did not alter the expression of total AMPK and LKB1 **(S1G Fig).** In contrast, inhibiting proteasomes using MG132 did not prevent the degradation of AMPK and LKB1 **(Fig 1K and 1L)** but prevented the degradation of IκB **(S1H Fig)**.

### Sirt1 is degraded upon *S*. Typhimurium infection

**Fig 2:**
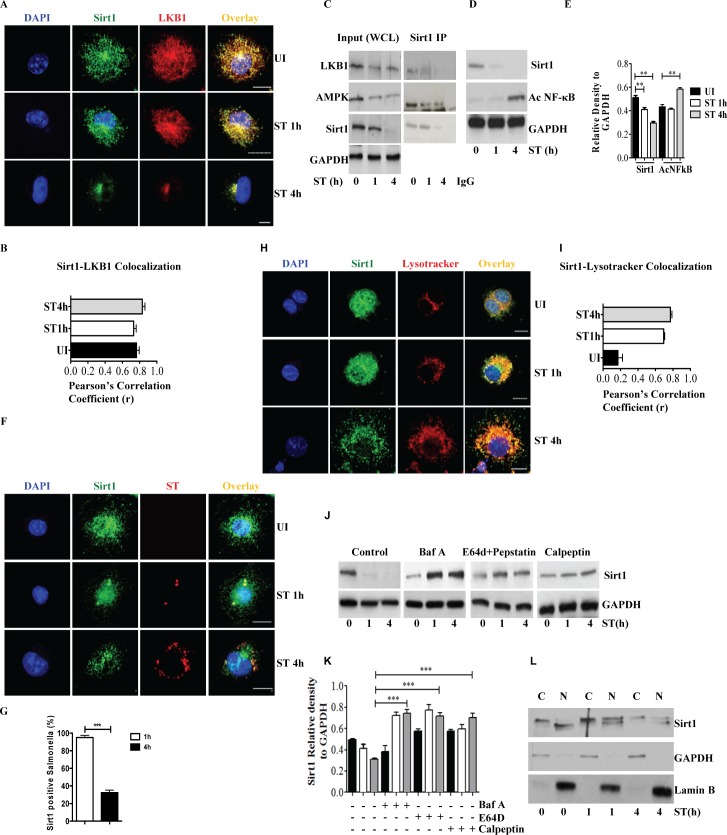
Sirt1 is degraded upon *S*. Typhimurium infection. **(A)** Confocal image of Sirt1 and LKB1 in BMDMs upon *S*. Typhimurium infection (n = 3). **(B)** Pearson’s correlation coefficient of Sirt1 with LKB1 calculated by measuring 42 ROIs. **(C)** Sirt1 was immunoprecipitated (IP) from uninfected and *S*. Typhimurium-infected BMDMs and the precipitated samples were immunoblotted (IB) for LKB1, AMPK and Sirt1 (n = 2). (**D**) Immunoblot of Sirt1, acetylated NFκB and GAPDH in BMDMs upon *S*. Typhimurium infection. Data shown are representative of 6 independent experiments. (**E**) Densitometric analysis of immunoblots. Bar graphs are expressed as mean ± SEM, ***p≤0.001 and **p≤0.01. (**F**) Immunofluorescence image of BMDMs stained for Sirt1 and *S*. Typhimurium (n = 4). **(G)** Quantitation of Sirt1-ST co-localization with SCVs. 100 SCVs were counted and expressed as percentage co-localization. Bar graphs are expressed as mean ± SEM, ***p≤0.001. (**H**) Sirt1-Lysotracker red co-localization in BMDMs upon *S*. Typhimurium infection (n = 4). **(I)** Pearson’s correlation coefficient of Sirt1 with Lysotracker red calculated by measuring minimum of 50 ROI. (**J**) Sirt1 expression upon *S*. Typhimurium infection in BMDMs treated with bafilomycinA (BafA), E64D, pepstatin A and calpeptin. **(K)** Sirt1 expression levels are quantified by densitometric analysis. Data shown are representative of 3 independent experiments. Bar graphs are expressed as mean ± SEM, ***p≤0.001 and **p≤0.01. **(L)** Sirt1 expression in nuclear (N) and cytoplasmic (C) fractions of BMDMs infected with *S*. Typhimurium. LaminB and GAPDH were used as housekeeping controls for nuclear and cytoplasmic fractions respectively (n = 2). Scale bar = 10μm for microscopical images.

Activation of LKB1 requires deacetylation by Sirt1 [17]. Immunofluorescence analysis showed that Sirt1 co-localized with LKB1 in uninfected cells, during the early (1h) and late phase of infection (4h) **(Fig 2A and 2B)**. We also found that LKB1 and AMPK co-immunoprecipitated with Sirt1, yet the abundance of the proteins were markedly reduced at 4h post infection **(Fig 2C)**. Immunoblot analysis confirmed that Sirt1 protein expression was downregulated in *S*. Typhimurium-infected macrophages **(Fig 2D and 2E)**. Notably, a significant change in the mRNA expression of Sirt1 was not observed **(S2A Fig)**, suggesting a post-translational mechanism by which *S*. Typhimurium downregulates Sirt1. Sirt1 co-localized with *S*. Typhimurium containing vacuoles (SCV) at 1h post infection, which diminished at 4h (**Fig 2F and 2G**). We also observed that Sirt1 and LKB1 co-localized on SCV shaped vesicles **(S2B Fig)** at 1h post infection. Immunoblot analysis of isolated *S*. Typhimurium-containing phagosomes revealed the presence of Sirt1 in phagosomes within 30min, which rapidly declined at later time points post infection **(S2C Fig)**. *S*. Typhimurium infection induced increased co-localization of Sirt1 with LysoTracker Red **(Fig 2H and 2I)** and LAMP1 **(S2D and S2E Fig)**, suggesting that degradation of Sirt1 is lysosome-mediated. We confirmed lysosomal degradation of Sirt1 by inhibiting lysosomal activity by bafilomycin A, E64D or calpeptin, all of which prevented Sirt1 degradation **(Fig 2J and 2K)**. Bafilomycin A treatment also prevented the degradation of AMPK and LKB1 **(S2F Fig)**. In contrast, degradation of Sirt1 was not prevented when treated with proteasome inhibitor MG132 **(S2G Fig)**. Heat-killed *S*. Typhimurium **(S2H Fig)** and LPS **(S2I Fig)** did not induce the degradation of Sirt1. These observations indicate that *S*. Typhimurium induces the translocation of Sirt1 along with AMPK and LKB1 to SCVs and lysosomes followed by degradation. Importantly, Sirt1 is known to shuttle between nucleus and cytoplasm, depending on the induced stress [19]. Analysis of cytoplasmic and nuclear fractions isolated from *S*. Typhimurium-infected macrophages revealed that cytosolic Sirt1 presented with a slightly higher molecular weight compared to that of the nuclear fraction in the uninfected cells **(Fig 2L)**. The shift in band size is probably brought about by phosphorylation of Sirt1 by kinases, which is a prerequisite for transport out of the nucleus mediated by CRM1 [27]. Indeed, inhibition of CRM1-mediated nuclear export by leptomycin-B reduced the translocation of Sirt1 to the cytosol and its degradation **(S2J and S2K Fig)** similar to the translocation of p53 which was examined as a positive control **(S2L Fig)**. Leptomycin treatment also reduced the activation and degradation of AMPK and LKB1 **(S2M Fig)**. Taken together, our data suggest that *S*. Typhimurium infection stimulates the nuclear export of Sirt1 onto lysosomes for degradation.

### *S*. Typhimurium triggered cytosolic translocation and degradation of Sirt1 involves AKT

**Fig 3:**
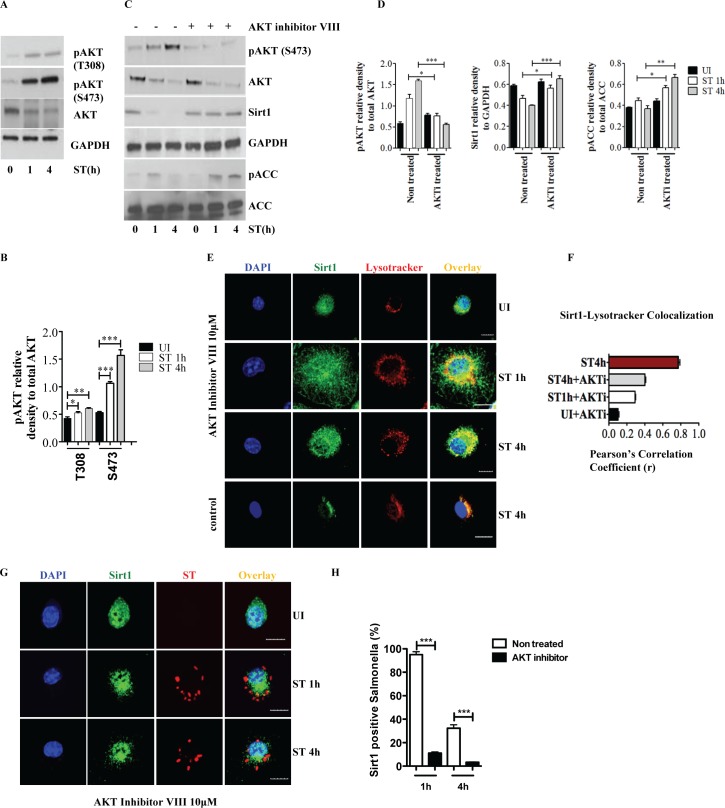
*S*. Typhimurium triggered cytosolic translocation and degradation of Sirt1 involves AKT. **(A)** Immunoblot analysis of AKT activation upon *S*. Typhimurium infection in BMDMs. **(B)** The phosphorylated and total AKT amounts are quantified by densitometric analysis. Data shown are representative of at least 3 independent experiments. Bar graphs are expressed as mean ± SEM, ***p≤0.001, **p≤0.01 and *p≤0.05. **(C)** Protein expression of AKT, Sirt1, GAPDH and ACC from BMDMs pretreated with or without AKT inhibitor VIII prior to infection with *S*. Typhimurium. Western bots are representative of 3 independent experiments. **(D)** The phosphorylated AKT, ACC and Sirt1 amounts are quantified by densitometric analysis. Bar graphs are expressed as mean ± SEM, ***p≤0.001, **p≤0.01 and *p≤0.05. (**E**) Confocal immunofluorescence image showing Sirt1-LysoTracker Red co-localization in BMDMs pretreated with AKT inhibitor VIII followed by *S*. Typhimurium infection. BMDMs untreated with AKT inhibitor VIII but infected with *S*. Typhimurium for 4h is shown for comparison (n = 3). **(F)** Pearson’s correlation coefficient of Sirt1 with LysoTracker Red calculated by measuring 35 ROIs. **(G)** Sirt1- *S*. Typhimurium co-localization in BMDMs pretreated with AKT inhibitor VIII followed by *S*. Typhimurium infection (n = 3). Scale bar = 10μm for microscopical images. **(H)** Quantitation of LysoTracker Red co-localization with SCVs. 100 SCVs were counted and expressed as percentage co-localization. Bar graphs are expressed as mean ± SEM, ***p≤0.001.

Sirt1 nucleocytoplasmic shuttling is regulated by PI3K-AKT signaling pathway [19]. *S*. Typhimurium infection enhanced the basal phosphorylation of AKT at S473 residue and to a minor extent at Thr308 **(Fig 3A and 3B)**, which is consistent with the idea that cytosolic translocation is mediated by AKT leading to subsequent lysosomal degradation of Sirt1. In addition, the AKT-mTOR pathway controls lysosomal function [28]. To examine whether AKT is involved in *S*. Typhimurium-induced Sirt1 degradation, macrophages were treated with AKT inhibitor VIII. AKT inhibition prevented the degradation of Sirt1 **(Fig 3C and 3D)**. Consistently, AKT inhibition led to increased AMPK activity as indicated by phosphorylation of ACC **(Fig 3C and 3D)**. Confocal microscopy showed that AKT inhibitor VIII treatment significantly reduced colocalization of Sirt1 with lysosomes **(Fig 3E and 3F)** and *S*. Typhimurium **(Fig 3G and 3H)**. Inhibition of PI3K, an upstream activator of AKT, also prevented Sirt1 degradation **(S3A Fig)**. These observations indicate that inactivation of AKT leads to stabilization of Sirt1 resulting in sustained AMPK activation during the later phase of *S*. Typhimurium infection.

### Increased mTOR activation leads to degradation of Sirt1 upon *S*. Typhimurium infection

**Fig 4:**
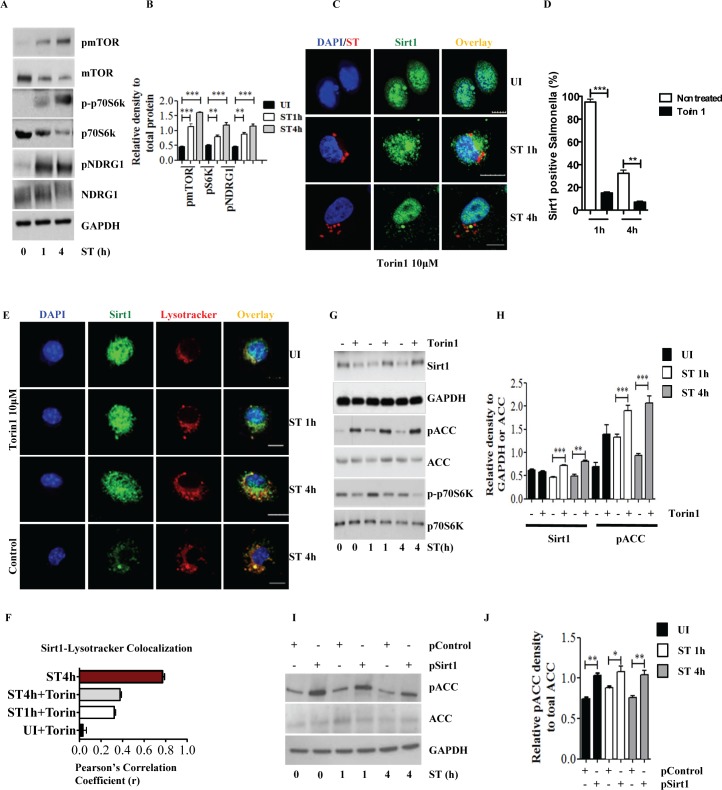
Increased mTOR activation leads to degradation of Sirt1 upon *S*. Typhimurium infection. (**A**) Immunoblot analysis of *S*. Typhimurium-infected BMDMs for mTOR and its downstream targets p70S6K and NDRG1. **(B)** Densitomertic analysis of phosphorylation amounts of mTOR, p70s6K and NDRG1. Data shown are representative of at least 3 independent experiments. Bar graphs are expressed as mean ± SEM, ***p≤0.001, **p≤0.01 and *p≤0.05. **(C)** Confocal image of Sirt1- *S*. Typhimurium. **(D)** Quantitation of LysoTracker Red co-localization with SCVs. 100 SCVs were counted and expressed as percentage co-localization. Bar graphs are expressed as mean ± SEM, ***p≤0.001. **(E)** Sirt1-LysoTracker Red co-localization in BMDMs pretreated with Torin1 followed by *S*. Typhimurium infection. Sirt1-LysoTracker Red co-localization in untreated-BMDMs infected with *S*. Typhimurium for 4h is shown for comparison (n = 3). **(F)** Pearson’s correlation coefficient of Sirt1 with LysoTracker Red calculated by measuring 35 selected regions of interest (ROI) using olympus fluoview fv1000 software. **(G)** Immunoblot analysis of Sirt1, ACC phosphorylation and S6Kinase activation in *S*. Typhimurium-infected BMDMs pretreated with Torin1. **(H)** Mean densitometric data of Sirt1 and phosphorylated ACC were analyzed and normalized to GAPDH and total ACC respectively (n = 3). Bar graphs are expressed as mean ± SEM, ***p≤0.001 and **p≤0.01. **(I)** Immunoblot of phosphorylated ACC in BMDMs transfected with control or Sirt1-expressing plasmids. Western blots are representative of three experiments. **(J)** Densitomertic analysis of phosphorylation amounts of ACC is shown from 3 independent experiments. Bar graphs are expressed as mean ± SEM, ***p≤0.001, **p≤0.01 and *p≤0.05.

Early activation of AKT is facilitated by SopB a virulence factor of *S*. Typhimurium. The question arises as to the mechanism by which *S*. Typhimurium activates AKT at a later time point. The pronounced phosphorylation of AKT at S473 **(Fig 3A)** suggested the involvement of mTOR. mTORC1 regulates vacuolar fission, which redistributes the luminal contents of phagosomes into the lysosome network [29]. Consistent with previous reports[4,11], we observed that *S*. Typhimurium infection increases the activity of both mTORC1 and mTORC2, indicated by phosphorylation of the well-established targets ribosomal S6 kinase (S6K) and N-myc downstream-regulated gene (NDRG1), respectively **(Fig 4A and 4B)**. Therefore, we investigated whether Sirt1 translocation on to SCVs and lysosomes is mTOR dependent. Indeed, *S*. Typhimurium-infected macrophages treated with Torin1 (inhibitor of both mTORC1 and mTORC2) significantly decreased the co-localization of Sirt1 with *S*. Typhimurium **(Fig 4C and 4D)**. Inhibition of mTOR also reduced Sirt1 translocation on to lysosomes **(Fig 4E and 4F)** and attenuated its degradation **(Fig 4G)**. Moreover, *S*. Typhimurium-phagosomes isolated from cells treated with Torin1 showed markedly reduced Sirt1 **(S4A Fig)**. As observed with AKT inhibition, mTOR inhibition also preserved AMPK-mediated phosphorylation of ACC **(Fig 4G and 4H)**. Similarly, ectopic expression of Sirt1 showed increased activity of AMPK **(Fig 4I and 4J)**. We conclude from our findings that *S*. Typhimurium–induced translocation and degradation of Sirt1 in phagolysosomes is mTOR and AKT dependent, which is crucially important for the disruption of Sirt1-dependent AMPK activation.

### *S*. Typhimurium evades autophagy by disrupting Sirt1-dependent AMPK activation

**Fig 5:**
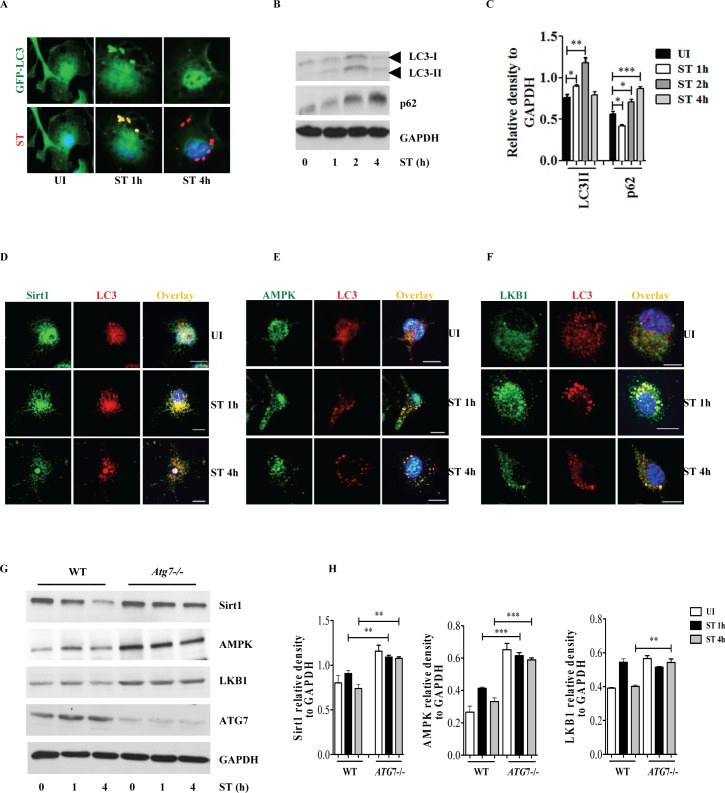
*S*. Typhimurium evades autophagy by disrupting Sirt1-dependent AMPK activation. **(A)** Immunofluorescence image of *S*. Typhimurium co-localization with LC3 in GFP-LC3 expressing BMDMs at indicated time points. Data shown are representative of 3 independent experiments (n = 3). **(B)** Immunoblot analysis of p62 and LC3 expression upon *S*. Typhimurium infection in BMDMs. **(C)** LC3 and p62 expression levels are quantified by densitometry analysis. Data shown are from 3 independent experiments. **(D)** Confocal image of macrophages stained for Sirt1 and LC3. **(E)** BMDMs stained for LC3 and AMPK upon *S*. Typhimurium infection. **(F)** Confocal image of macrophages stained for LKB1 and LC3. **(G)** Immunoblot analysis of Sirt1, AMPK and LKB1 in wild type (WT) and Atg7-deficient macrophages. **(H)** Densitometric analysis of Sirt1, AMPK and LKB1 immunoblots (n = 3). Bar graphs are expressed as mean ± SEM, ***p≤0.001, **p≤0.01 and *p≤0.05. Scale bar = 10μm for microscopical images.

Sirt1, AMPK and mTOR are critically involved in the regulation of autophagy, which is an important cell-autonomous defense mechanism required for pathogen clearance [30]. The biphasic activation and inactivation of Sirt1 and AMPK raised the question about the consequences for autophagy. As has been shown in HeLa cells [4,11], infection of macrophages isolated from LC3-GFP expressing transgenic mice revealed that localization of LC3 on SCVs occurred only at the early time point (1h p.i.) tested **(Fig 5A)**. LC3-GFP on SCVs was significantly decreased at 4h **(Fig 5A)**. Concomitantly, conversion of LC3I to II was observed at 1h post infection **(Fig 5B and 5C)**. Notably, p62, which is a *bona fide* target of autophagosomal degradation declined at 1h to accumulate at 2h and 4h post infection, indicating that the autophagic flux was initially increased and subsequently impaired indicating a short and transient phase of autophagy in *S*. Typhimurium-infected cells **(Fig 5B and 5C)**. As degradation of AMPK and LKB1 involves lysosomes rather than the proteasome (**Fig 1K and 1L)**, we tested whether Sirt1, AMPK and LKB1 are targeted to lysosomes via autophagy. Microscopical examinations revealed that Sirt1 **(Fig 5D and S5A Fig)**, AMPK **(Fig 5E and S5B Fig)** and LKB1 **(Fig 5F and S5C Fig)** co-localized with LC3. Furthermore, Sirt1, AMPK and LKB1 accumulated in autophagy deficient macrophages derived from Atg7^fl/fl^ LysMcre^+/+^ mice (Atg7^-/-^) **(Fig 5G and 5H)**. These data suggest that transient induction of autophagy is sufficient to target Sirt1, AMPK and LKB1 for lysosomal degradation.

The impact of AMPK degradation on the termination of autophagy in *S*. Typhimurium-infected macrophages was confirmed by pharmacological activation of AMPK using AICAR. As expected AICAR highly upregulated autophagy as assessed by LC3 conversion and p62 degradation **(S5D and S5E Fig)**. An increase in the co-localization of LC3 with SCVs at 4h post-infection was also observed **(S5F and S5G Fig)**. These data suggest that *S*. Typhimurium suppresses autophagy upstream of AMPK.

### *S*. Typhimurium-mediated targeting of Sirt1 for lysosomal degradation is virulence-dependent

**Fig 6:**
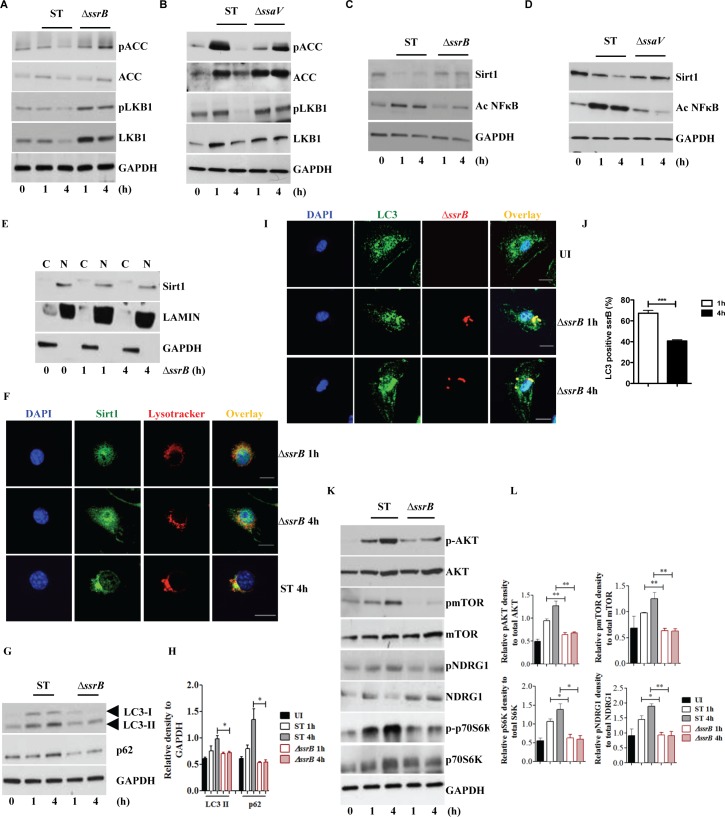
*S*. Typhimurium mediated targeting of Sirt1 for lysosomal degradation is virulence dependent. Immunoblot analysis of ACC and LKB1 activation upon infection with *ΔssrB* **(A)** and *ΔssaV* **(B)** compared to *S*. Typhimurium. Sirt1 and acetylated-NFκB from macrophages infected with *ΔssrB* **(C)** *and ΔssaV* **(D)** compared to *S*. Typhimurium. **(E)** Expression of Sirt1 from cytoplasmic (C) and nuclear (N) fraction from BMDMs infected with *ΔssrB*. **(F)** Confocal image of Sirt1 and LysoTracker Red in *ΔssrB*-infected BMDMs. Sirt1-LysoTracker Red co-localization in untreated BMDMs infected with *S*. Typhimurium for 4h is shown for comparison (n = 3). **(G)** Immunoblot analysis of LC3 and p62. **(H)** Densitometric analysis of LC3 lipidation and p62 (n = 4). **(I)** Immunofluorescence image of *ΔssrB*-infected BMDMs stained for LC3 and LPS of *S*. Typhimurium (n = 3). **(J)** Quantitation of LC3 co-localization with SCVs. 100 SCVs were counted and expressed as percentage co-localization. **(K)** AKT, mTOR, p70S6K, NDRG1 expression upon S. Typhimurium (ST) and *ΔssrB* infection in BMDMs. **(L)** Densitometric analysis of AKT, mTOR, p70S6K and NDRG1 are shown from 3 independent experiments. Bar graphs are expressed as mean ± SEM, ***p≤0.001. Scale bar = 10μm for microscopical images.

**Fig 7:**
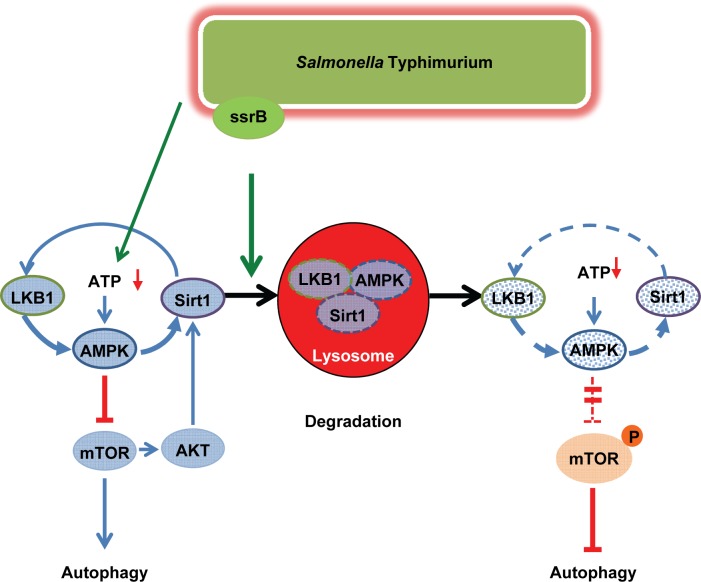
Schematic representation. SsrB-regulated virulence proteins of S. Typhimurium impedes Sirt1-LKB1-AMPK circuitry network to evade autophagy. S. Typhimurium induces energy depletion resulting in transient activation of AMPK. AMPK-mediated inhibition of mTOR and induction of autophagy are blunted by SPI2-regulated effector proteins by targeting Sirt1/LKB1/AMPK complex for lysosomal degradation.

Previous reports suggested that mTOR-dependent AKT activation is dependent on virulence factors of *S*. Typhimurium [31]. Therefore we investigated whether the degradation of Sirt1 and subsequent inhibition of AMPK activation and autophagy could be virulence dependent. To address this we used *ΔssrB* and *ΔssaV* mutants of *S*. Typhimurium. SsrB is a response regulator of a two-component system that regulates the majority of the SPI2 encoded virulence factors [32] and SsaV is a component of the SPI2 type III secretion apparatus [33,34]. Infection of macrophages with *ΔssrB* **(Fig 6A and S6A Fig)** or *ΔssaV* **(Fig 6B and S6B Fig)** resulted in prolonged phosphorylation of ACC indicative of sustained AMPK activation. Similarly, the *S*. Typhimurium mutants, *ΔssrB* **(Fig 6C and S6C Fig)** and *ΔssaV* **(Fig 6D and S6D Fig)** failed to induce Sirt1 degradation and preserved the enzymatic activity of Sirt1. Analysis of nuclear and cytoplasmic fractions of macrophages infected with *ΔssrB* showed reduced translocation of Sirt1 to the cytoplasm **(Fig 6E)** and subsequent targeting to lysosomes **(Fig 6F and S6E Fig)**. Notably, infection with the *S*. Typhimurium mutants, *ΔssrB* **(Fig 6G and 6H)** and *ΔssaV* **(S6F and S6G Fig)** resulted in increased LC3 conversion and reduced p62 expression indicating ongoing autophagy and unhampered autophagic flux, respectively. Indeed, the *ΔssrB* **(Fig 6I and 6J)** and *ΔssaV* **(S6H and S6I Fig)** mutants also co-localized with LC3 at 4h post-infection, indicating that autophagy was not impaired. Consistently, both mutants failed to activate mTOR suggesting that mTOR activation and attenuation of autophagy are SsrB and SsaV dependent **(Fig 6K and 6L and S6J and S6K Fig)**. Taken together, the results suggest that *S*. Typhimurium employs SsrB-dependent virulence factors of SPI2 to disrupt the Sirt1/LKB1/AMPK checkpoint of mTOR and autophagy **(Fig 7)**.

## Discussion

Intracellular survival and replication within eukaryotic host cells is a hallmark of *S*. Typhimurium, which is sensed as a major virulence factor of Salmonella. After internalization by phagocytes, *Salmonella* remains in a specific membrane-bound compartment, termed Salmonella-containing vacuole (SCV). By means of a type III secretion system (T3SS) encoded by Salmonella pathogenicity island 2 (SPI2), *S*. Typhimurium translocates a number of effector proteins into the cytosol that interfere with host cell defense mechanisms to avoid fusion of SCV with lysosomes and eventually bacterial killing. We here report a novel function of SPI2 which targets the AMPK-dependent activation pathway of mTOR, a prominent checkpoint of cellular homeostasis that modulates a wide array of critical cellular functions, including proliferation, metabolism, and survival. *S*. Typhimurium infection of macrophages resulted in early energy loss, which is immediately sensed by AMPK. Activated AMPK down-regulates mTOR, which in turn initiates a cellular stress response including autophagy. Our data reveal Sirt1 and LKB1 as essential members of a cytosolic AMPK activation complex, which are targeted by *S*. Typhimurium for lysosomal degradation in a SPI2 dependent manner. The physical dismantling of the AMPK activation complex allowed robust mTOR activation and subsequent cease of autophagy.

Numerous studies have elucidated the significance of autophagy in the cell autonomous defense against *S*. Typhimurium [5,35,36]. However, the regulation of autophagy in macrophages during *S*. Typhimurium infection is not well understood. Initiation of autophagy depends on the activation status of mTOR, which senses the intracellular nutrient availability. It was shown recently that *S*. Typhimurium induces transient depletion of amino acids in HeLa cells leading to transient activation of autophagy. However, amino acids were gradually replenished resulting in activation of mTOR and inhibition of autophagy [11]. mTOR forms two functionally distinct complexes, mTORC1 and mTORC2, the activities of both being dependent on the activation of mTOR by AKT within the complex [9]. *S*. Typhimurium virulence factor SopB was shown previously to activate AKT at Ser473 in an mTORC2-dependent manner at an early time point [11,31,37]. In agreement with these reports, we observed an increase in phosphorylation of AKT at Ser473. Moreover, it has been demonstrated that activation of AKT and mTOR is regulated by focal adhesion kinase in a SPI2 dependent manner [38]. Consistently, our results with the *ΔssrB* and *ΔssaV S*. Typhimurium mutants now indicate that the sustained activation of AKT and mTOR is dependent on *S*. Typhimurium virulence factors encoded by SPI2 and/or the type III secretion apparatus. Increased activation of mTOR and AKT are both known to result in the inhibition of autophagy, initiated at early time periods of infection. Notably, AMPK enhances autophagy by at least two routes, that is, by mTOR-independent mechanisms, including the phosphorylation of Ulk1 at Ser317 and Ser777 [39] yet also by inhibiting mTOR through phosphorylation of TSC2 to promote the formation of an Ulk1-Atg13-FIP200 complex [40]. We here demonstrate that *S*. Typhimurium infection is associated with early but transient activation of AMPK secondary to rapid loss of ATP. Whereas the early drop in ATP led to an increase in the activity of AMPK, *S*. Typhimurium induced targeting of the AMPK-activation complex for lysosomal degradation reduced AMPK activity during the later phase of infection despite sustained low levels of ATP.

A major observation of this study revealed that lysosomal targeting of AMPK and its subsequent degradation is dependent on *S*. Typhimurium SPI2, as shown by the *ΔssrB S*. Typhimurium mutant and SPI2-type III secretion defective mutant *ΔssaV* [34]. Transient AMPK activation in *S*. Typhimurium-infected cells resulted in ineffective autophagy with no signs of autophagic flux indicated by accumulation of p62. In contrast, pharmacological activation of AMPK using AICAR increased LC3 conversion and p62 degradation, suggesting that autophagic flux is highly dependent on sustained AMPK activation, which was counteracted by *S*. Typhimurium in infected macrophages. In general, *S*. Typhimurium survives in macrophages and establishes systemic infection by employing genes encoded on SPI2 [41,42,43]. SsrB is part of a two-component system that specifically activates multiple SPI2 localized genes, which are predominantly expressed after the SCV is acidified [32] and SsaV is a component of the type III secretion apparatus that injects the SPI2 virulence factors into the host cell [33]. Our study reveals that SPI2 encoded virulence factors dismantle an important cellular defense mechanism by targeting Sirt1/LKB1/AMPK complex for lysosomal degradation.

AMPK activation is primarily regulated by the upstream kinase LKB1 [26]. We observed that LKB1 constitutively colocalized with AMPK, which is consistent with previous reports that LKB1 activates AMPK. Notably, the cytosolic localization of LKB1 depends on its previous deacetylation by Sirt1 in the nucleus. Sirt1-mediated deacetylation of nuclear LKB1 enables the export of the kinase to the cytosol, where it is phosphorylated by the protein kinase Czeta [17]. Whereas the activation of AMPK by Sirt1 has been studied in the context of mitochondrial metabolism [18], the regulation of Sirt1 during host-pathogen interactions is not well understood. We show here that *S*. Typhimurium markedly down-regulates Sirt1 expression commencing within 1h post infection. Several lines of evidence indicated that *S*. Typhimurium induces lysosomal degradation of Sirt1, which is consistent with previous observations that Sirt1 is cleaved by cathepsins in endothelial progenitor cells during stress induced premature senescence [1]. Whereas the translocation of Sirt1 onto SCVs results in subsequent lysosomal degradation, *S*. Typhimurium seems to be able to escape into the cytosol thereby avoiding lysosomal degradation. The decline in Sirt1 expression upon *S*. Typhimurium infection was accompanied by inhibition of AMPK. Indeed, ectopic overexpression of Sirt1 restored AMPK activity, suggesting that Sirt1 is essentially required for the activation of AMPK during *S*. Typhimurium infection. Apart from its role in regulating AMPK with secondary effects on autophagy, Sirt1 has been reported to directly regulate autophagy by deacetylating Atg5 and Atg7 [44]. Thus, *S*. Typhimurium through initiating lysosomal degradation of Sirt1 disrupts autophagic defense mechanisms at several molecular levels.

## Materials and methods

### Ethics statement

All animal procedures were in accordance with guidelines laid out by the German Animal Welfare Act and were approved by the North Rhine-Westphalian State Agency for Nature, Environment, and Consumer Protection [Landesamt für Natur, Umwelt and Verbraucherschutz (LANUV) Nordrhein-Westfalen; File no: 84–02.05.40.14.082 and 84–02.04.2015.A443] and the University of Cologne.

### Mice and generation of bone marrow derived macrophages

Bone marrow derived macrophages (BMDMs) were prepared as described [14] from C57BL/6J mice maintained and bred in the animal facility of Center for Molecular Medicine, University of Cologne. Atg7^fl/fl^ LysMcre^+/+^ myeloid specific Atg7 knockout mice were a kind gift from Michael Schramm, University of Cologne. Mice were sacrificed by cervical dislocation and bone marrows from the femurs were flushed using RPMI medium. The flushed cells were centrifuged and resuspended in RPMI containing 10% FBS. Cells were seeded in tissue culture dishes and allowed to differentiate into macrophages in medium supplemented with 20% L929 cell-culture supernatant for 7 days. Non-adherent cells were removed on days 2 and 4, and adherent macrophages were used from day 7 onwards.

### Infection of macrophages

Macrophages were infected as described. In brief, cells were seeded into tissue culture plates and infected with *S*. Typhimurium (SL1344), *S*. Typhimurium mutants; *ΔssaV* or *ΔssrB* (MOI, 10). After 30 min, extracellular bacteria were removed and cells were incubated for 2h in medium containing 50μg/ml gentamicin and then were washed and subsequently cultured in medium containing less gentamicin (10μg/ml). At desired time points cells were collected for analysis. *S*. Typhimurium mutant *ΔssrB* generated in the lab of Brett Finlay was obtained from Subash Sad. *S*. Typhimurium mutant *ΔssaV* was obtained from the lab of Ivan Dikic.

### Drug treatment and transfection

The inhibitors and activators were used 2h prior to infection until unless otherwise mentioned. bafilomycin A1 (100nM), E64d/pepstatin A (10μg/ml), calpeptin (10μg/ml), AKT inhibitor VIII (10μM), leptomycin B (50nM), Torin1 (10μM), AICAR (1mM), MG132 (10μM) and wortmanin (1μM). For plasmid transfection, WT Sirt1 plasmid created in the laboratory of Toren Finkel was procured from Addgene (cat no: 10962) [45]. Transfection of plasmid was done using jetPEI transfection reagent (Polyplus-transfection) following manufacturer’s instructions.

### Chemicals and antibodies

LysoTracker deep red (L12492), Superscript III first strand synthesis system (18080–051), ProLong Gold antifade reagents with DAPI (P36935), Goat-anti-rabbit alexafluor 488 (A-11034), 594 (A-11072), Goat-anti-mouse alexafluor 488 (A-11017), 594 (A-11020), Image-iT FX signal enhancer (I36933) were obtained from Life technologies. Bafilomycin A1 (B1793), concanamycin A (27689), MG132 (M8692), lactacystin (L6785), pepstatin A (P5318), leptomycin B (L2913), AICAR (A9978) and antibody for LC3 (L7543) were obtained from Sigma Aldrich. E64d (sc-201280), calpeptin (117591-20-5) and antibodies for Sirt1 (sc-15404), LAMP1 (sc-17768), Lamin B (sc-6217) and Syntaxin 3 (sc-393518) were purchased from Santacruz. Protease inhibitor tablets (88666), BCA Protein Assay Kit (23227), NEPER nuclear and cytoplasmic extraction kit (78833), anti-LPS of *Salmonell*a Typhimurium (MA1-83451) and formaldehyde (28908) were obtained from Thermo Scientific. Antibodies for SIRT1 (3931), phospho-NF-κB p65 (3033), NF-κB p65 (4764), Acetyl- NF-κB p65 (3045), phospho-AMPK (2535), AMPKα (2532), phospho-acetyl-CoA Carboxylase (3661), acetyl-CoA carboxylase (3662), phospho AKT-T308 (2965), phospho AKT-S473 (4060), AKT (4691), phospho-p70S6 kinase (9205), p70S6 kinase (9202), SQSTM1/p62 (5114), phospho-4E-BP1 (9455), 4E-BP1(9452), phospho-NDRG1 (3217), phospho-mTOR (2974), mTOR (2972), phospho-LKB1 (3482), LKB1 (3047) were purchased from Cell Signaling and antibody against GAPDH (AF5718) was procured from R&D systems. Light Cycler 480 SYBR Green I Master (04707516001) from Roche. RNeasy mini Kit (74106), RNase free DNase set (79254) and DNAseI (79254) from Qiagen.

### ATP and NAD measurements

ATP measurements were performed at Metabolomic Discoveries, Berlin. Metabolites from *S*. Typhimurium-infected macrophages were extracted using an extraction buffer supplied by the company and the extract was analyzed using LC-QTOF mass spectrometer. Sample concentrations were adjusted to optimally detect ATP. ATP levels were also estimated in our laboratory using Cell Titer-Glo Luminescent Cell Viability Assay (Promega) following manufacturer’s instructions. The Intracellular NAD levels upon infection were measured using NAD^+^/NADH Assay Kit (Abcam, San Francisco, CA) according to manufacturer's instructions.

### Immunostaining and microscopy

BMDMs were grown on 12mm coverslips (0.1-0.2x10^6^ cells at the time of treatment or infection). At desired time points, the coverslips were washed with PBS and cells were fixed with 4% (wt/vol) formaldehyde for 15min at room temperature. The fixed cells were washed three times with PBS and permeabilized with 0.3% tritonX-100 in PBS for 5 minutes at room temperature. The cells were washed with PBS followed by incubation with Image-iT FX signal followed by incubation with primary antibodies for overnight. The cells were then incubated with appropriate secondary antibodies labelled with Alexa flour 488 or 594. The coverslips were mounted on glass slides using ProLong Gold antifade containing DAPI. Cells were imaged using an inverted Confocal microscope (Olympus IX81 equipped with Cell^R Imaging Software; Tokyo, Japan) using a 60x Plano Apo oil objective with 1.45 numerical aperture. Pearson’s correlation was calculated using Olympus fluoview fv1000 software.

### Phagosome preparation

Phagosome preparation was done as previously described [46]. A minimum of 10x10^6^ of BMDMs was seeded on to 10cm dishes followed by infection. After desired time points, the cells were washed with PBS and incubated with equilibration buffer (50 mM Pipes buffer, pH7.0; 50 mM KCl; 2 mM MgCl2; 5 mM EGTA; 1 mM DTT and 10 μM cytochalasin B) on ice for 20min. After incubation, lysis buffer was added (50 mM Pipes buffer, pH7.0; 50 mM KCl; 2 mM MgCl2; 5 mM EGTA; 220 mM mannitol; 68 mM sucrose; 1 mM DTT and 10 μM cytochalasin B) and lysed cells were scraped using a cell scrapper and collected in a tube. The macrophage lysate was passed 15 times through a 23G needle for homogenization and spun down at 400g for 5 min. The post nuclear supernatant was adjusted to 35% (wt/vol) by addition of 65% sucrose in HEPES/EGTA buffer. A sucrose gradient was prepared by overlaying 1ml of HEPES/EGTA buffer containing 65% sucrose, 2ml of 55% sucrose, 3ml of 32.5% sucrose and 3ml of 10% sucrose. The gradient was centrifuged at 28,500 rpm for 1h at 4°C and the phagosomal fraction at the interface between 55%-39% was harvested. The phagosomal fraction was diluted with HEPES buffer and centrifuged further at 28,500 rpm for 1h at 4°C and the pellet was lysed with RIPA buffer and used for western blot analysis.

### Immunoprecipitation

Cells were lysed with radio-immunoprecipitation assay (RIPA) buffer containing protease inhibitors. After clearing the cell lysate with protein A/G agarose beads (Millipore) for an hour, the beads were removed by centrifugation and the whole cell lysate (approximately 500μg of protein) was treated with 4 μg of antibody against Sirt1 for 18h. Protein G agarose beads were then added and incubated for an additional 1hr. The immunoprecipitated proteins along with the agarose beads were collected by centrifugation. The collected beads were washed several times with RIPA buffer. The washed samples were mixed with SDS-PAGE sample loading buffer, boiled and resolved on a 10% SDS-polyacrylamide gel and the respective proteins precipitated were identified by western blotting.

### Western blotting

Western blotting was performed on proteins extracted using RIPA buffer. BCA was done to quantify the amount of proteins in the lysates. Required samples were mixed 1:1 with 2X sample loading buffer, boiled at 95°C and resolved by SDS-PAGE. Proteins were then transferred on to a PVDF membrane blocked with 5% milk or BSA and probed with the primary antibody of interest followed by treatment with an appropriate secondary antibody conjugated to horseradish peroxidase. The blots were developed using an enhanced chemiluminescence substrate (GE Health sciences) and bands were identified by exposing the membrane on to an X-ray film. Densitometric analysis of immunoblots was performed using NIH ImageJ.

## Supporting information

S1 Fig **(A)** Intracellular levels of ATP in BMDMs upon *S*. Typhimurium infection quantified using cellTiter-glo luminescence kit. Bar graphs are expressed as mean ± SEM, ***p≤0.001 (n = 5). **(B)** Confocal image showing AMPK-LAMP1. **(C)** Pearson’s correlation coefficient of AMPK with LAMP1 calculated by measuring 25 regions of interest (ROI) using olympus fluoview fv1000 software. **(D)** LKB1-LAMP1 in BMDMs upon *S*. Typhimurium infection (n = 3). Scale bar represents 5μm for microscopy images. **(E)** Pearson’s correlation coefficient of AMPK with LAMP1 calculated by measuring 25 regions of interest (ROI) using olympus fluoview fv1000 software. **(F)** Cell lysates of heat-killed *S*. Typhimurium (HKST)-infected BMDMs were immunoblotted for Sirt1 and GAPDH. **(G)** Immunoblot analysis of p62 with and without concanamycinA. **(H)** IκBα levels upon MG132 treatment upon *S*. Typhimurium infection. Immunoblots are representative of 2 independent experiments.
(TIF)

S2 Fig **(A)** mRNA transcript levels of *sirt1* from BMDMs infected with *S*. Typhimurium were analyzed by qRT-PCR at indicated time points (n = 3). **(B)** Confocal image of Sirt1 and LKB1 upon *S*. Typhimurium infection. **(C)** Immunoblot of phagosomal fractions from BMDMs infected with *S*. Typhimurium at indicated time points were immunoblotted for Sirt1 and syntaxin3A (protein loading control for phagosomes) (n = 3). **(D)** Immunofluorescence image of Sirt1 and LAMP1 in BMDMs upon *S*. Typhimurium infection (n = 2). **(E)** Pearson’s correlation coefficient of AMPK with LAMP1 calculated by measuring 25 selected regions of interest (ROI) using olympus fluoview fv1000 software. **(F)** Western blot analysis of AMPK and LKB1 on cell lysates of *S*. Typhimurium-infected BMDMs pretreated with bafilomycin A. **(G)** Cell lysates of *S*. Typhimurium-infected BMDMs pretreated with proteosomal inhibitor MG132 were immunoblotted for Sirt1 and GAPDH. **(H)** Cell lysates of heat-killed-*S*. Typhimurium (HKST) infected BMDMs were immunoblotted for Sirt1 and GAPDH. **(I)** Cell lysates of LPS-treated BMDMs were immunoblotted for Sirt1 and GAPDH. **(J)** Immunofluorescence image of BMDMs pretreated with leptomycin B and infected with *S*. Typhimurium stained for Sirt1 and *S*. Typhimurium (n = 3). **(K)** Cell lysates of BMDMs pretreated with leptomycin B and infected with *S*. Typhimurium were immunoblotted for Sirt1 and GAPDH. **(L)** Confocal images of p53 localization from non-treated and leptomycin B treated BMDMs upon *S*. Typhimurium infection. **(M)** Cell lysates of BMDMs pretreated with leptomycin B and infected with *S*. Typhimurium were immunoblotted for pAMPK, AMPK, pLKB1, LKB1 and GAPDH. Scale bar = 10μm for microscopical images.
(TIF)

S3 Fig Cell lysates of BMDMs pretreated with wortmannin and infected with *S*. Typhimurium were immunoblotted for Sirt1 and GAPDH. (TIF)

S4 Fig Western blot analysis of isolated phagosomes from BMDMs pretreated with Torin1 and infected with *S*. Typhimurium for Sirt1 and syntaxin3A. Total cell lysates were probed for GAPDH.
(TIF)

S5 Fig **(A)** Pearson’s correlation coefficient of Sirt1 and LC3 co-localization calculated by measuring at least 25 ROIs using olympus fluoview fv1000 software. **(B)** Pearson’s correlation coefficient of AMPK and LC3 co-localization calculated by measuring at least 25 ROIs using olympus fluoview fv1000 software. **(C)** Pearson’s correlation coefficient of LKB1 with LC3 co-localization calculated by measuring 32 ROIs using olympus fluoview fv1000 software. **(D)** Immunoblot of LC3 and p62 from AICAR-pretreated BMDMs followed by *S*. Typhimurium infection at indicated times. Western blots are representative of three experiments. Scale bar represents 5μm for microscopy images. **(E)** Densitomertic analysis of LC3 and p62 are shown from 3 independent experiments. **(F)** Immunofluorescence image of *S*. Typhimurium-infected BMDMs treated with AICAR stained for LC3 and *S*. Typhimurium. Untreated BMDMs infected with *S*. Typhimurium for 4h is shown for comparison (n = 3). **(G)** Quantitation of LC3 co-localization with SCVs. 100 SCVs were counted and expressed as percentage co-localization. Scale bar for microscopical images = 10μm.
(TIF)

S6 Fig Densitomertic analysis of phosphorylated ACC and LKB1 in macrophages infected with *ΔssrB* **(A)** or *ΔssaV* **(B)** and compared to *S*. Typhimurium-infected macrophages. Densitomertic analysis of Sirt1 and acetylated NFκB expression in macrophages infected with *ΔssrB* **(C)** or *ΔssaV* **(D)** compared to *S*. Typhimurium-infected macrophages. Data shown are from 3 independent experiments. **(E)** Pearson’s correlation coefficient of Sirt1 colocalization with LysoTracker Red upon *ΔssrB* infection was calculated by measuring 35 selected regions of interest (ROI) using olympus fluoview fv1000 software. **(F)** Immunoblot analysis of LC3 and p62 upon infection with *ΔssaV*. **(G)** Densitometric analysis of LC3 lipidation and p62 (n = 3). **(H)** Immunofluorescence image of *ΔssaV* and *S*. Typhimurium-infected BMDMs stained for LC3 and LPS of *S*. Typhimurium (n = 3). **(I)** 100 SCVs were counted and expressed as percentage co-localization. **(J)** Phosphorylation of AKT and mTOR upon ST and *ΔssaV* infection in BMDMs. **(K)** Densitometric analysis of phosphorylated AKT and mTOR are shown from 3 independent experiments. Scale bar = 10μm for microscopical images. Bar graphs are expressed as mean ± SEM, ***p≤0.001, **p≤0.01 and *p≤0.05.
(TIF)
