# Supplementary figures and images for: Salmonella Typhimurium disrupts Sirt1/AMPK checkpoint control of mTOR to impair autophagy
Source: PLoS Pathog. 2017 Feb 13;13(2):e1006227. doi: 10.1371/journal.ppat.1006227 (PMC5325604; doi:10.1371/journal.ppat.1006227)

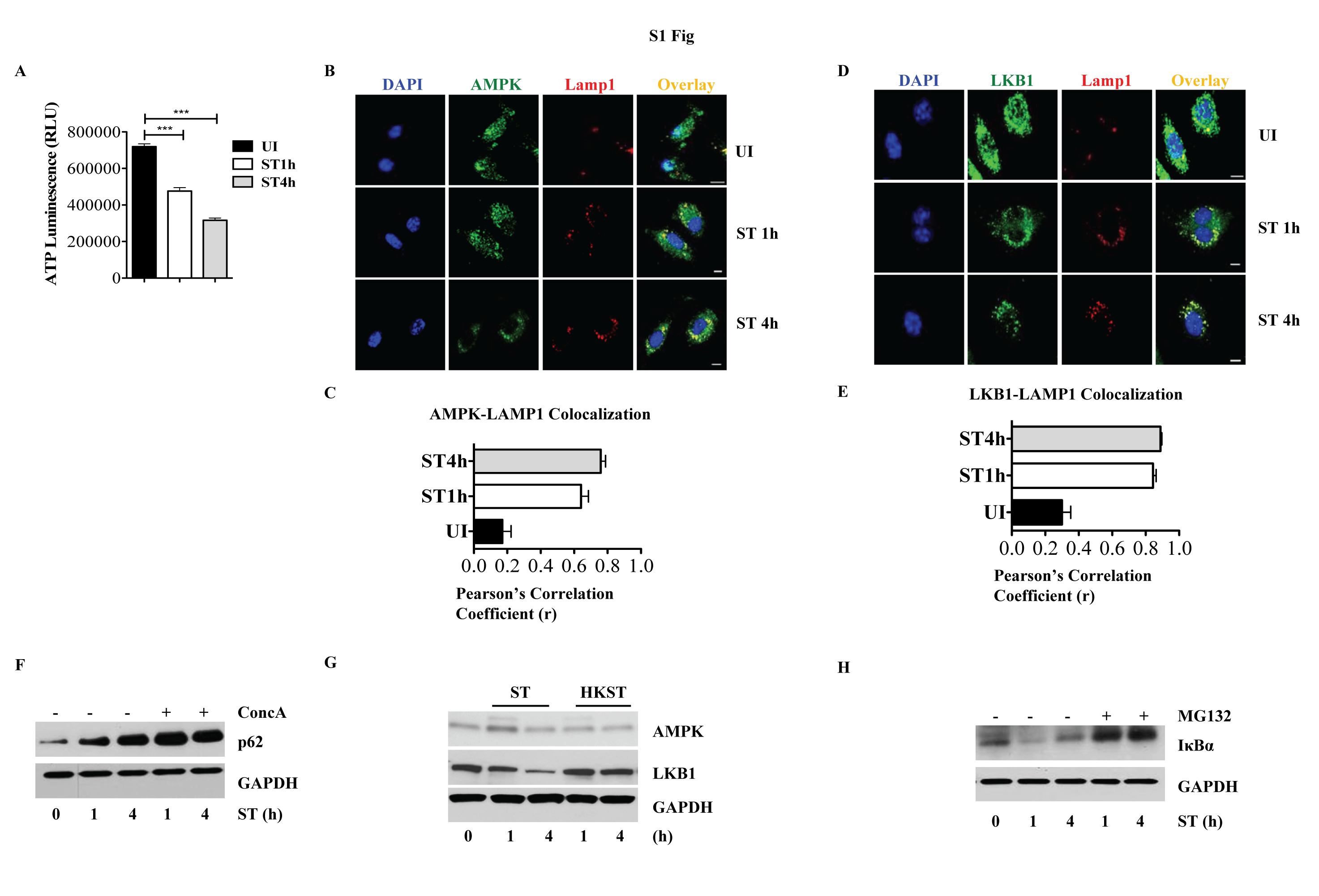

Supplement: S1 Fig — (A) Intracellular levels of ATP in BMDMs upon S. Typhimurium infection quantified using cellTiter-glo luminescence kit. Bar graphs are expressed as mean ± SEM, ***p≤0.001 (n = 5). (B) Confocal image showing AMPK-LAMP1. (C) Pearson’s correlation coefficient of AMPK with LAMP1 calculated by measuring 25 regions of interest (ROI) using olympus fluoview fv1000 software. (D) LKB1-LAMP1 in BMDMs upon S. Typhimurium infection (n = 3). Scale bar represents 5μm for microscopy images. (E) Pearson’s correlation coefficient of AMPK with LAMP1 calculated by measuring 25 regions of interest (ROI) using olympus fluoview fv1000 software. (F) Cell lysates of heat-killed S. Typhimurium (HKST)-infected BMDMs were immunoblotted for Sirt1 and GAPDH. (G) Immunoblot analysis of p62 with and without concanamycinA. (H) IκBα levels upon MG132 treatment upon S. Typhimurium infection. Immunoblots are representative of 2 independent experiments. (TIF) [file ppat.1006227.s001.tif]

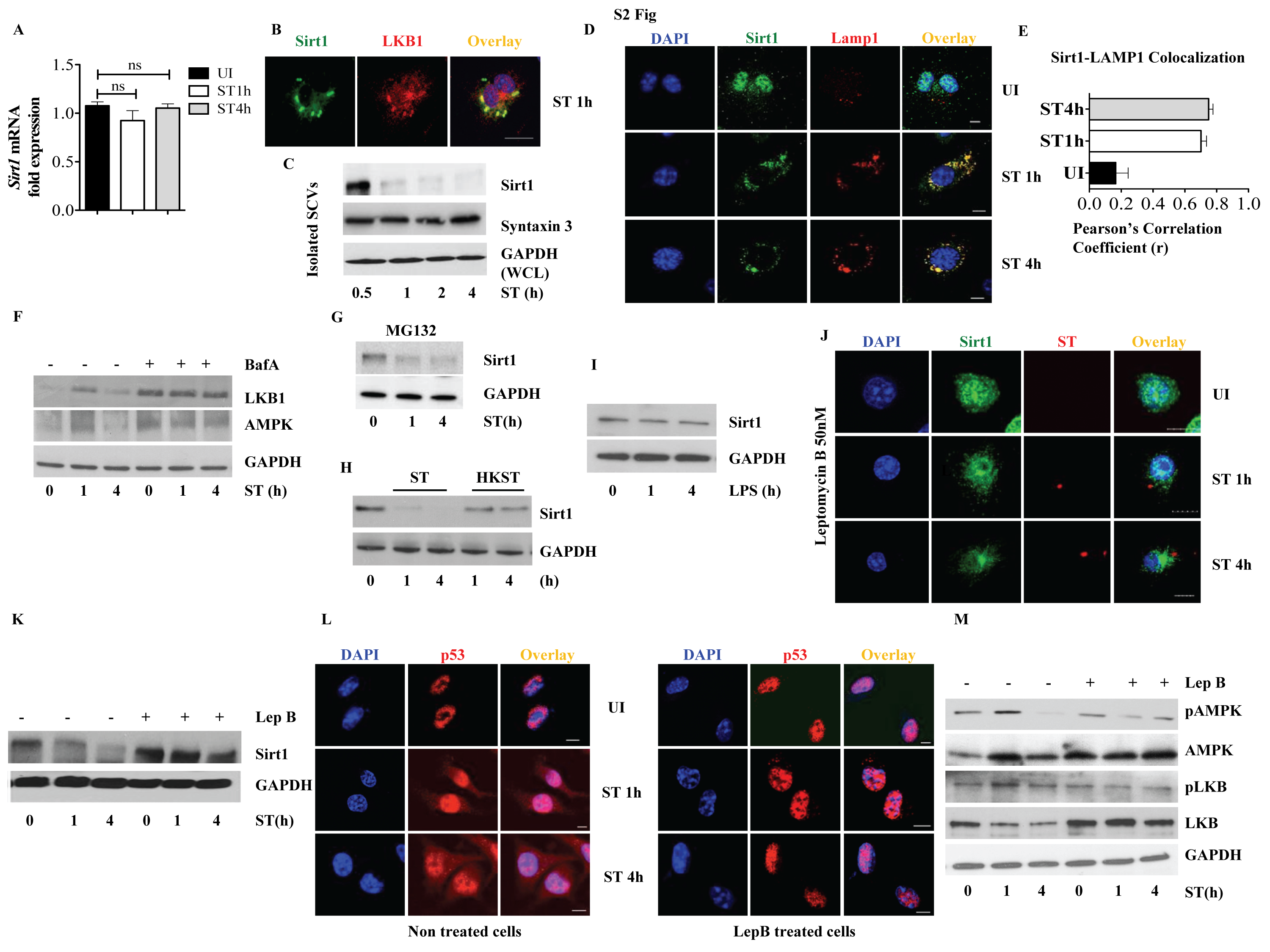

Supplement: S2 Fig — (A) mRNA transcript levels of sirt1 from BMDMs infected with S. Typhimurium were analyzed by qRT-PCR at indicated time points (n = 3). (B) Confocal image of Sirt1 and LKB1 upon S. Typhimurium infection. (C) Immunoblot of phagosomal fractions from BMDMs infected with S. Typhimurium at indicated time points were immunoblotted for Sirt1 and syntaxin3A (protein loading control for phagosomes) (n = 3). (D) Immunofluorescence image of Sirt1 and LAMP1 in BMDMs upon S. Typhimurium infection (n = 2). (E) Pearson’s correlation coefficient of AMPK with LAMP1 calculated by measuring 25 selected regions of interest (ROI) using olympus fluoview fv1000 software. (F) Western blot analysis of AMPK and LKB1 on cell lysates of S. Typhimurium-infected BMDMs pretreated with bafilomycin A. (G) Cell lysates of S. Typhimurium-infected BMDMs pretreated with proteosomal inhibitor MG132 were immunoblotted for Sirt1 and GAPDH. (H) Cell lysates of heat-killed-S. Typhimurium (HKST) infected BMDMs were immunoblotted for Sirt1 and GAPDH. (I) Cell lysates of LPS-treated BMDMs were immunoblotted for Sirt1 and GAPDH. (J) Immunofluorescence image of BMDMs pretreated with leptomycin B and infected with S. Typhimurium stained for Sirt1 and S. Typhimurium (n = 3). (K) Cell lysates of BMDMs pretreated with leptomycin B and infected with S. Typhimurium were immunoblotted for Sirt1 and GAPDH. (L) Confocal images of p53 localization from non-treated and leptomycin B treated BMDMs upon S. Typhimurium infection. (M) Cell lysates of BMDMs pretreated with leptomycin B and infected with S. Typhimurium were immunoblotted for pAMPK, AMPK, pLKB1, LKB1 and GAPDH. Scale bar = 10μm for microscopical images. (TIF) [file ppat.1006227.s002.tif]

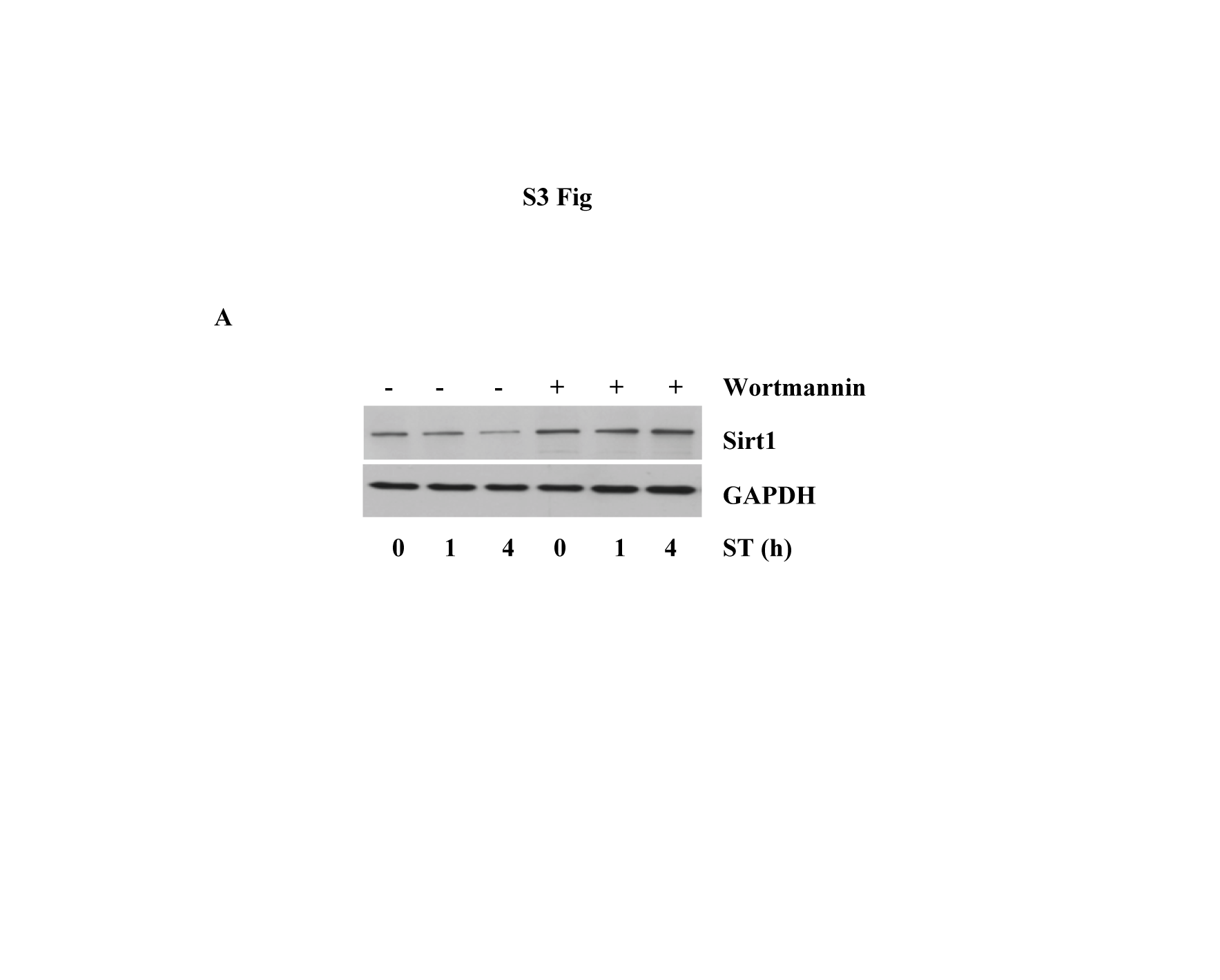

Supplement: S3 Fig — (TIF) [file ppat.1006227.s003.tif]

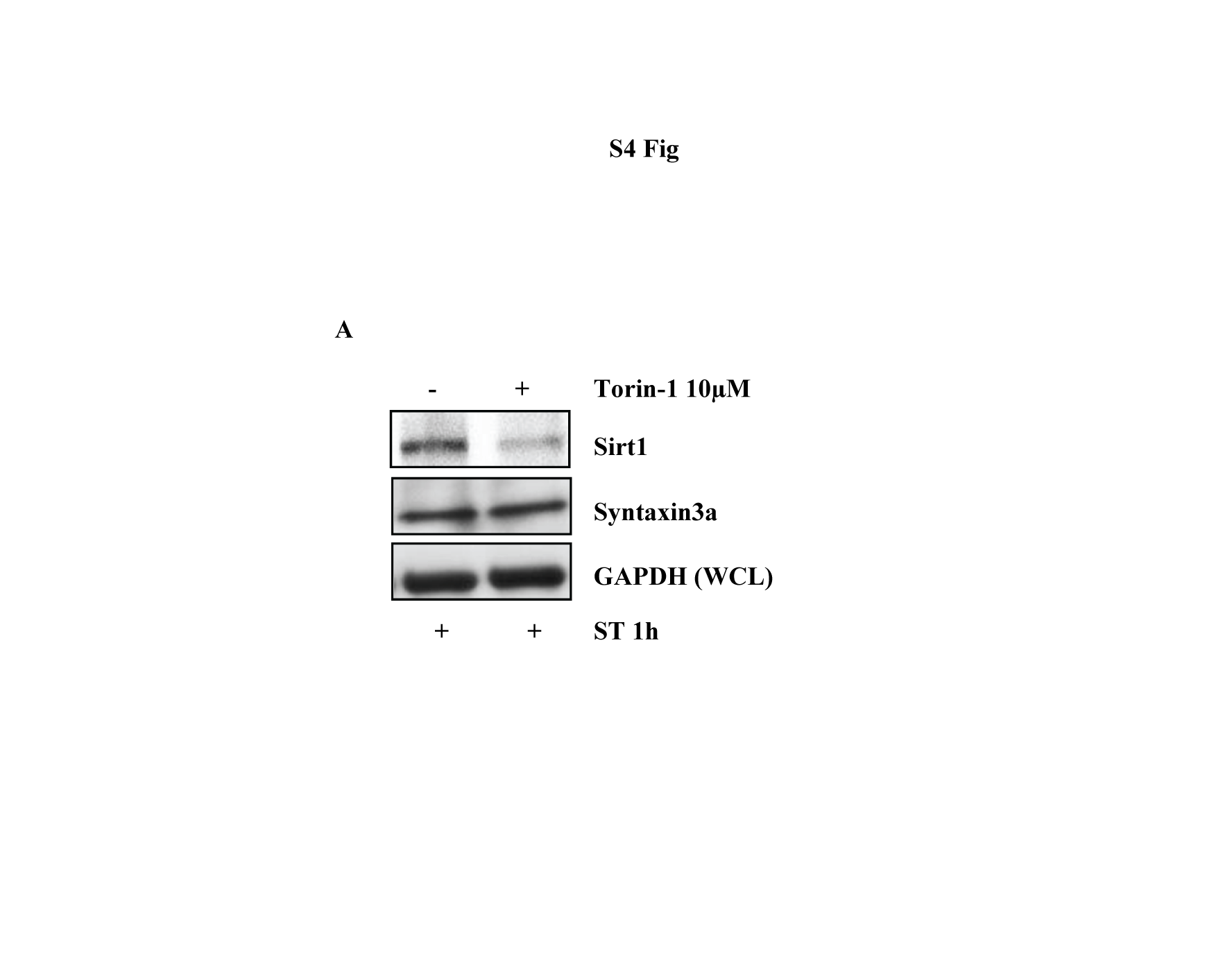

Supplement: S4 Fig — Total cell lysates were probed for GAPDH. (TIF) [file ppat.1006227.s004.tif]

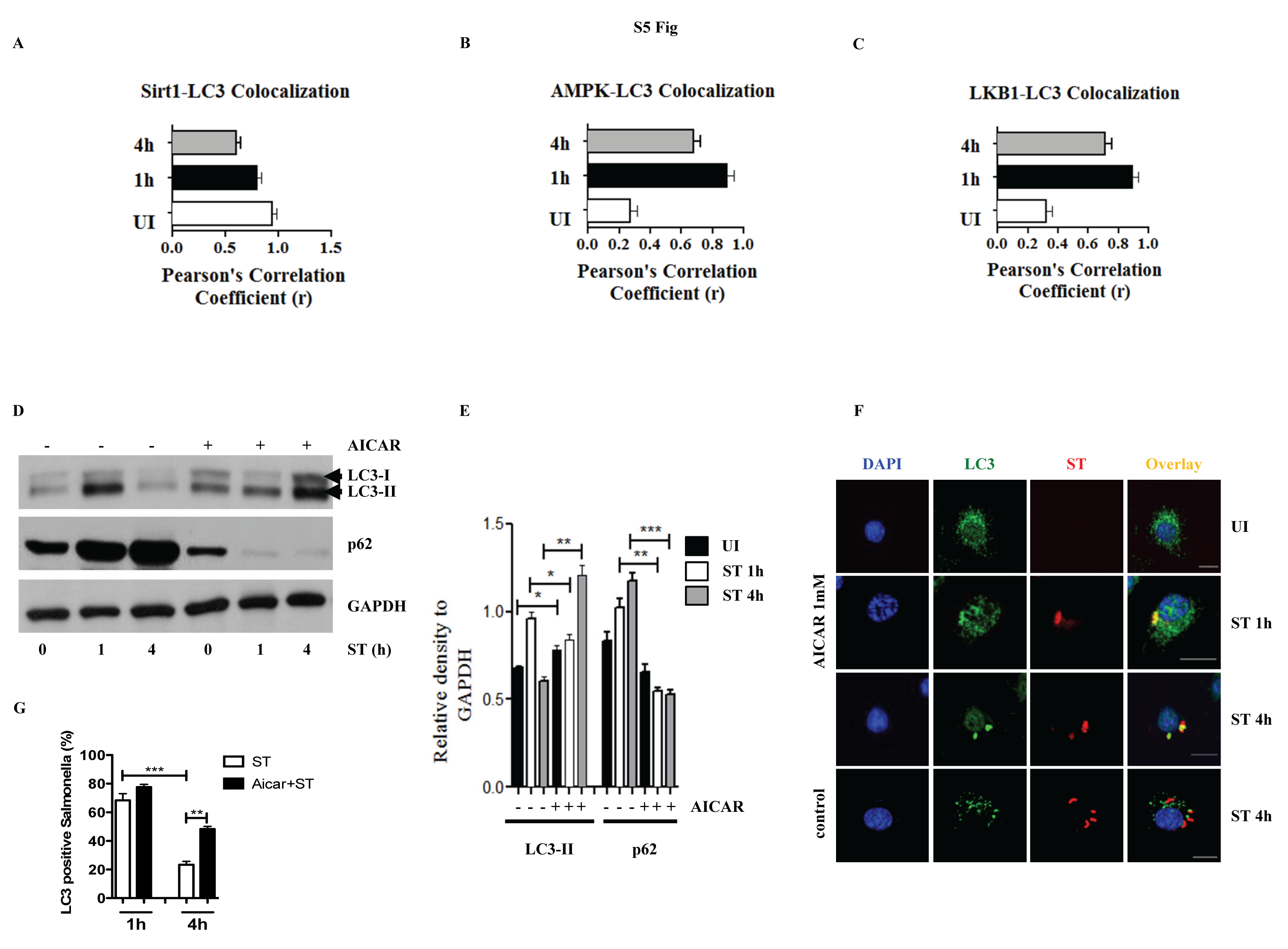

Supplement: S5 Fig — (A) Pearson’s correlation coefficient of Sirt1 and LC3 co-localization calculated by measuring at least 25 ROIs using olympus fluoview fv1000 software. (B) Pearson’s correlation coefficient of AMPK and LC3 co-localization calculated by measuring at least 25 ROIs using olympus fluoview fv1000 software. (C) Pearson’s correlation coefficient of LKB1 with LC3 co-localization calculated by measuring 32 ROIs using olympus fluoview fv1000 software. (D) Immunoblot of LC3 and p62 from AICAR-pretreated BMDMs followed by S. Typhimurium infection at indicated times. Western blots are representative of three experiments. Scale bar represents 5μm for microscopy images. (E) Densitomertic analysis of LC3 and p62 are shown from 3 independent experiments. (F) Immunofluorescence image of S. Typhimurium-infected BMDMs treated with AICAR stained for LC3 and S. Typhimurium. Untreated BMDMs infected with S. Typhimurium for 4h is shown for comparison (n = 3). (G) Quantitation of LC3 co-localization with SCVs. 100 SCVs were counted and expressed as percentage co-localization. Scale bar for microscopical images = 10μm. (TIF) [file ppat.1006227.s005.tif]

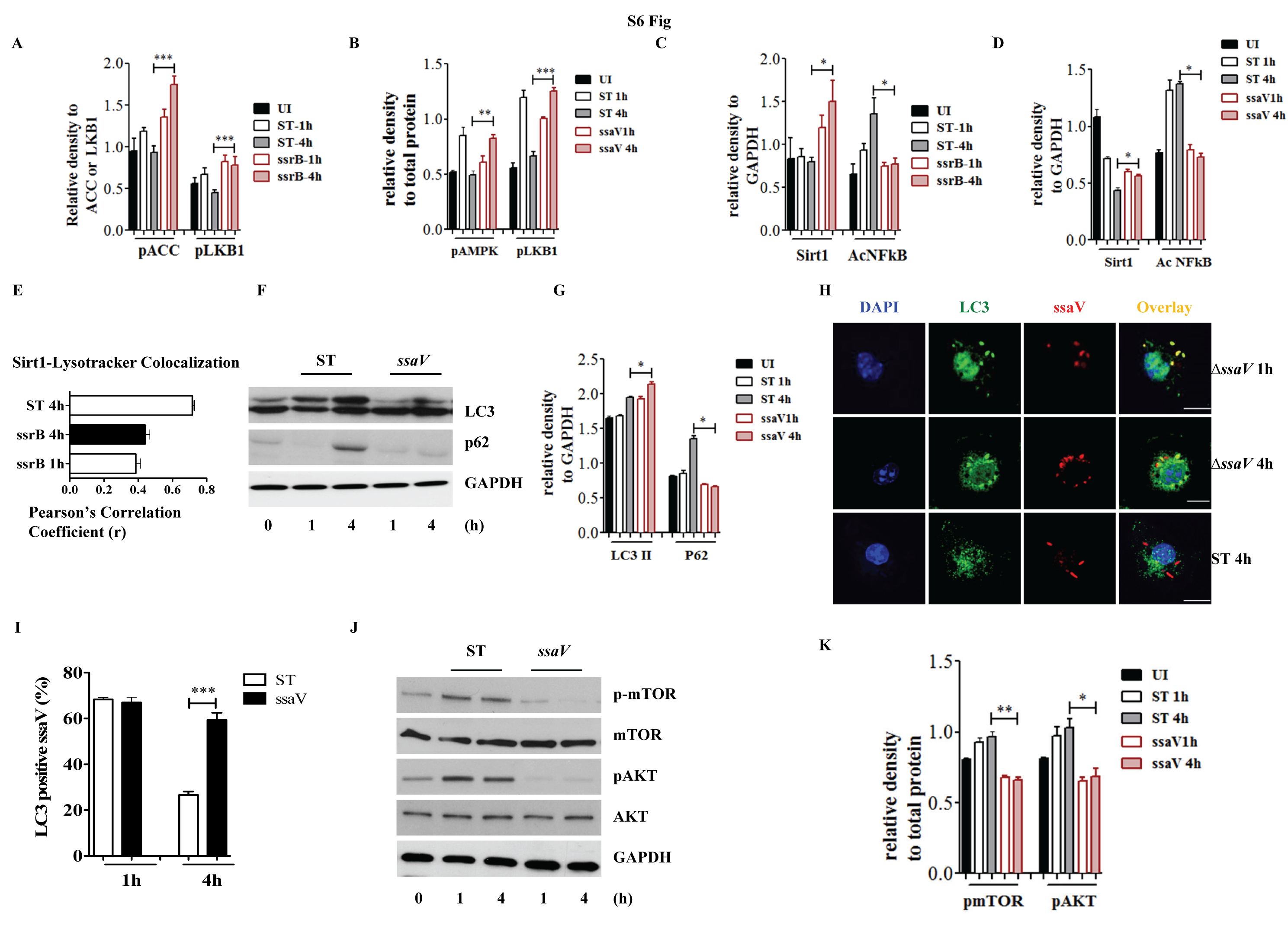

Supplement: S6 Fig — Densitomertic analysis of phosphorylated ACC and LKB1 in macrophages infected with ΔssrB (A) or ΔssaV (B) and compared to S. Typhimurium-infected macrophages. Densitomertic analysis of Sirt1 and acetylated NFκB expression in macrophages infected with ΔssrB (C) or ΔssaV (D) compared to S. Typhimurium-infected macrophages. Data shown are from 3 independent experiments. (E) Pearson’s correlation coefficient of Sirt1 colocalization with LysoTracker Red upon ΔssrB infection was calculated by measuring 35 selected regions of interest (ROI) using olympus fluoview fv1000 software. (F) Immunoblot analysis of LC3 and p62 upon infection with ΔssaV. (G) Densitometric analysis of LC3 lipidation and p62 (n = 3). (H) Immunofluorescence image of ΔssaV and S. Typhimurium-infected BMDMs stained for LC3 and LPS of S. Typhimurium (n = 3). (I) 100 SCVs were counted and expressed as percentage co-localization. (J) Phosphorylation of AKT and mTOR upon ST and ΔssaV infection in BMDMs. (K) Densitometric analysis of phosphorylated AKT and mTOR are shown from 3 independent experiments. Scale bar = 10μm for microscopical images. Bar graphs are expressed as mean ± SEM, ***p≤0.001, **p≤0.01 and *p≤0.05. (TIF) [file ppat.1006227.s006.tif]
